# Editorial: Mental Imagery in Clinical Disorders

**DOI:** 10.3389/fpsyt.2017.00049

**Published:** 2017-03-27

**Authors:** David G. Pearson, Julie Krans

**Affiliations:** ^1^Anglia Ruskin University, Cambridge, UK; ^2^KU Leuven, Leuven, Belgium

**Keywords:** mental imagery, clinical disorders, clinical psychology, eye movements, intrusive cognitions

**Editorial on the Research Topic**

**Mental Imagery in Clinical Disorders**

Mental imagery refers to the simulation or recreation of perceptual experience across different sensory modalities (1, 2). The study of mental imagery has clinical relevance because such imagery has been increasingly shown to play a key role across various psychological disorders (3, 4). The current research topic presents a diverse range of cutting-edge papers focusing on investigating the underlying mechanisms and/or treatment interventions associated with mental imagery in clinical disorders. We are very pleased with the final result, which comprises 15 articles drawn from the fields of psychiatry, psychology, and neuroscience. This topic provides a unique collection of articles that combine different perspectives from the field of clinical psychology with more diverse perspectives drawn from the wider literature on mental imagery.

One central theme across the topic is articles that have focused on the role of mental imagery in memories for negative events. Over the last decade, there has been a considerable impact in this area of, for example, working memory theory and dual task methodology [e.g., Ref. (5, 6)] on procedures associated with reducing intrusive cognitions. Two articles consider the potential limitations of this approach. van Veen et al. report two studies that examine how the speed of eye movement (EM) interacts with the emotionality and vividness of negative memories. Although a high rate of EM was associated with significant reduction in emotionality and vividness, contrary to expectation, the rate of EM did not interact with the extent of reported image vividness. The study reported by van Schie et al. examines the effect of EM on the retrieval of cued negative images acquired through word–image paired association. Dual-task EMs were found to have no effect on both self-rated vividness and emotionality of the images or latency responses. These studies highlight the importance of achieving greater understanding of the complex relationship between increased working memory load and selective interference effects on trauma-related imagery.

The research article by Nelis et al. takes a different but equally important approach by examining the recall of positive rather than negative life events. Two studies are reported in which participants are asked to retrieve positive autobiographical memories followed by induction of either image- or verbal-based processing styles. A concrete/imagery-based processing style was associated with a larger increase in positive effect in comparison to verbal processing, which is consistent with the perspective that mental imagery can serve as an “emotional amplifier” of personal memories (7).

In the Hypothesis and Theory article by Clark and MacKay, they propose a framework in which mental imagery is just one component part of an intrusive memory, with others including the autobiographical trauma memory itself, the process of involuntary recall, negative emotions, and attention hijacking. Mental imagery is identified as playing a key role in bridging the experience, memory, and intrusive recollection elements associated with a traumatic event. The opinion article by Strange and Takarangi also discusses the role played by mental imagery in trauma memory, but this time within the context of memory distortion for traumatic events. They argue that mental imagery contributes to source monitoring failures during retrieval, in which intrusive mental images contribute new false details, which over time become assimilated into people’s memory for the trauma event itself. Both of these theoretical contributions offer an important reminder that the strong sensory–perceptual nature of intrusive recollection should not be interpreted as a literal “replaying” of perceptual experience encoded during original trauma, and that memory intrusions need not be synonymous with the trauma memory itself.

A second theme represented in the topic explores the contribution of mental imagery across more diverse clinical disorders than those associated with trauma memory. Kearns and Engelhard’s study required students with public speaking anxiety to complete a script-driven imagery procedure depicting a future public speaking scenario. Eye movements performed concurrently while participants held the image of the scenario in mind significantly reduced heart rate in comparison to a control condition, suggesting that EM’s can produce a psychophysiological reduction in experienced emotion for future-oriented fearful events (“flash-forwards”).

In the opinion article by Klein and Moritz, they argue that intrusive mental imagery lies on a continuum where the boundary between experiences associated with clinical disorders and those experienced by the wider population is not clearly defined. They discuss the findings from recent online studies investigating major depressive disorder (MDD) and obsessive–compulsive disorder (OCD), which find 1 in 5 patients with OCD and 1 in 10 patients with mild-to-moderate MDD report strong to extreme mental imagery associated with their pathological cognitions. The opinion article by May et al. focuses on the Elaborated Intrusion Theory (EI), originally developed as a theoretical framework to account for substance-related craving, but here considered in relation to understanding clinical disorders such as PTSD and suicidal imagery. They argue that EI theory can provide a “roadmap” to help treat mental imagery of maladaptive goals across a range of different clinical disorders. This approach includes identifying those situations associated with greater risk for temptation and loss of control and methods for reducing attention to intrusive imagery such as mindfulness or acceptance training.

A further theoretical framework is discussed by Cili and Stopa who apply the self-memory system (SMS) theory of autobiographical memory (8) to understand how intrusive imagery can help maintain psychological disorders. They argue a critical way in which intrusive imagery maintains disorders through their representation and relationship with a patient’s sense of self, particularly through the “working self” component of the SMS framework. They also propose that therapeutic techniques, such as imagery rescripting, may be beneficial specifically because they are thought to target patients’ self-images and their associated meaning. The discussion of intrusive imagery is further broadened in the piece by Moran et al. who consider the importance of motor imagery in clinical disorders. They discuss the theoretical perspective that the brain is a dynamic predictive system that uses imagery-based simulation to integrate imagination, perception, and action processes, and they apply this framework in the context of clinical disorders such as PTSD, personality disorders, and social anxiety disorder. This article is a valuable reminder of the importance of non-visual imagery processes to clinical disorders.

A third theme in the research topic is articles that focus on imagery in healthy populations but have direct relevance to understanding clinical disorders. Hagenaars et al. present two experiments on healthy participants that examine whether mental imagery as a pre-trauma manipulation can influence fear bradycardia during subsequent viewing of affective pictures. Their findings are important for demonstrating that a highly automatic defense behavior such as bradycardia can successfully be influenced by pre-trauma mental imagery manipulations.

Intrusive memory processes in non-clinical groups are explored in articles by Krans et al. and Mace. Krans et al. report a questionnaire study conducted on undergraduate students assessing the quality, type, content, and potential function of involuntary cognitions experienced during the preceding month. Their findings show that, in relation to the subjective experience of involuntary cognitions by individuals, the specific subtype of cognition (i.e., fantasy, memory, and rumination) appears less important than its valence or content. The opinion article by Mace summarizes his work on involuntary autobiographical memory chains, in which the involuntary retrieval of one memory triggers other additional memories to quickly spring to mind. Mace’s theoretical account has been very useful in understanding how contextual priming of involuntary trauma memory might occur [e.g., Ref. (9, 10)].

The opinion articles by Missbach et al. and Kemps and Tiggemann both discuss the function of mental imagery in the context of food consumption and food cravings. Missbach et al. argue that research on mental imagery can help better understand the eating behavior and also aid the design of more effective individual-level interventions. It can also help explain why some individuals are more successful self-regulators in their consumption of food than others. In a similar vein, Kemps and Tiggemann discuss the growing evidence that mental imagery plays a key role during the subjective experience of food craving, including an evaluation of imagery-based craving reduction techniques. These techniques use modality-specific cognitive tasks to interfere with mental imagery and help suppress associated craving. This field of research is valuable in relation to understanding mental imagery in clinical disorders as it helps suggest new avenues to explore for effective clinical interventions for treating involuntary imagery-based cognitions.

In conclusion, we believe that the range of submissions presented in this research topic make a strong contribution to help in establishing a theoretical and methodological foundation that can enable the effective comparison of imagery across different disorders and across different domains. There are informative parallels that can be drawn between the literature on clinical disorders and current theoretical models that assign a functional role for intrusive imagery during craving and addiction. Mental imagery processes may also underlie the effectiveness of clinical interventions such as imagery rescripting, imaginal exposure in Cognitive Behavioral Therapy, schema-focused therapy, and cognitive bias modification training. Nonetheless, there still remains an ongoing need for further systematic comparison of the role of imagery across different disorders, with the goal of establishing those common elements, which are most relevant to investigate mental imagery within the context of clinical psychology.

## Author Contributions

All authors drafted the manuscript and provided critical revisions. All authors approved the final version of the manuscript for submission.

## Conflict of Interest Statement

The authors declare that the research was conducted in the absence of any commercial or financial relationships that could be construed as a potential conflict of interest.
